# The Structure Basis of Phytochemicals as Metabolic Signals for Combating Obesity

**DOI:** 10.3389/fnut.2022.913883

**Published:** 2022-06-13

**Authors:** Xiaoping Li, Liufeng Zheng, Bing Zhang, Ze-Yuan Deng, Ting Luo

**Affiliations:** State Key Laboratory of Food Science and Technology, Nanchang University, Nanchang, China

**Keywords:** phytochemicals, obesity, transcription factors, structure, metabolic signals

## Abstract

The consumption of phytochemicals, bioactive compounds in fruits and vegetables, has been demonstrated to ameliorate obesity and related metabolic symptoms by regulating specific metabolic pathways. This review summarizes the progress made in our understanding of the potential of phytochemicals as metabolic signals: we discuss herein selected molecular mechanisms which are involved in the occurrence of obesity that may be regulated by phytochemicals. The focus of our review highlights the regulation of transcription factors toll like receptor 4 (TLR4), nuclear factor (erythroid-derived 2)-like 2 (Nrf2), the peroxisome proliferator-activated receptors (PPARs), fat mass and obesity-associated protein (FTO) and regulation of microRNAs (miRNA). In this review, the effect of phytochemicals on signaling pathways involved in obesity were discussed on the basis of their chemical structure, suggesting molecular mechanisms for how phytochemicals may impact these signaling pathways. For example, compounds with an isothiocyanate group or an α, β-unsaturated carbonyl group may interact with the TLR4 signaling pathway. Regarding Nrf2, we examine compounds possessing an α, β-unsaturated carbonyl group which binds covalently with the cysteine thiols of Keap1. Additionally, phytochemical activation of PPARs, FTO and miRNAs were summarized. This information may be of value to better understand how specific phytochemicals interact with specific signaling pathways and help guide the development of new drugs to combat obesity and related metabolic diseases.

## Introduction

Obesity, normally results from an imbalance between energy consumption and energy expenditure, is one of the most prevalent problems challenging public health today. Obesity increases the risk of diseases including type 2 diabetes (1), cardiovascular disease and some types of cancer (2). However, no effective therapy has been developed for the treatment of obesity except for exercise and dietary regimes. Pharmacological treatment has been used for the long-term treatment of severely obese patients. Currently, orlistat, the world's only weight loss drug approved by the US Food and Drug Administration, has been used by some obese people (3). However, its application is limited for the side effects such as diarrhea, and vomiting (4). Therefore, the search for novel food and food compound interventions as a monotherapy or along with existing therapies is a main focus for nutritionists (5).

Phytochemicals, bioactive compounds in fruits and vegetables are produced through primary or secondary metabolism. They may be synthesized to protect plants from a variety of stresses, such as pathogenic attacks, predators, and UV irradiation (6). In recent years, there have been many studies on the regulation of signaling pathways by phytochemicals, most of which aim at the biological activity and physiological function of phytochemicals; however, little attention has been paid to the underlying molecular mechanism and preferred structure of phytochemicals for pathway activation.

In this review, the structure-activity relationship of phytochemicals on obesity and related chronic diseases were discussed. We summarize the progress made in our understanding of the anti-obesity effect of phytochemicals and their mechanisms of action: the effect of phytochemicals on transcription factors and some related molecular pathways. We focus on the transcription factors toll like receptor 4 (TLR4), peroxisome proliferator-activated receptors (PPARs), and transcription factors nuclear factor (erythroid-derived 2)-like 2 (Nrf2). Additionally, the fat mass and obesity-associated protein (FTO), and microRNA (miRNA) regulation of gene expression are also discussed.

## Toll Like Receptor 4 (TLR4) Signaling Pathway

Toll-like receptors (TLRs) are a family of pattern-recognition receptors (PRR) that trigger innate immune and inflammatory responses in response to invading microorganisms and non-microbial endogenous molecules (7). TLR4, one of the thirteen TLRs identified in mammals, influences symptoms induced by high fat diet-induced obesity, including insulin resistance (8–10), inflammation (8, 11, 12), and hepatic lipid accumulation (13–15).

TLR4 molecular structure includes a leucine-rich repeat (LRR) domain in the ectodomain, which is involved in the recognition of pathogen-associated molecular patterns (PAMPs), and a Toll/interleukin-1 receptor homology (TIR) domain in the cytoplasm, in which highly conserved cysteine residues are located (16). In addition to this cytoplasmic conserved region, other cysteine residues also reside in the extracellular domain.

**Figure 1:**
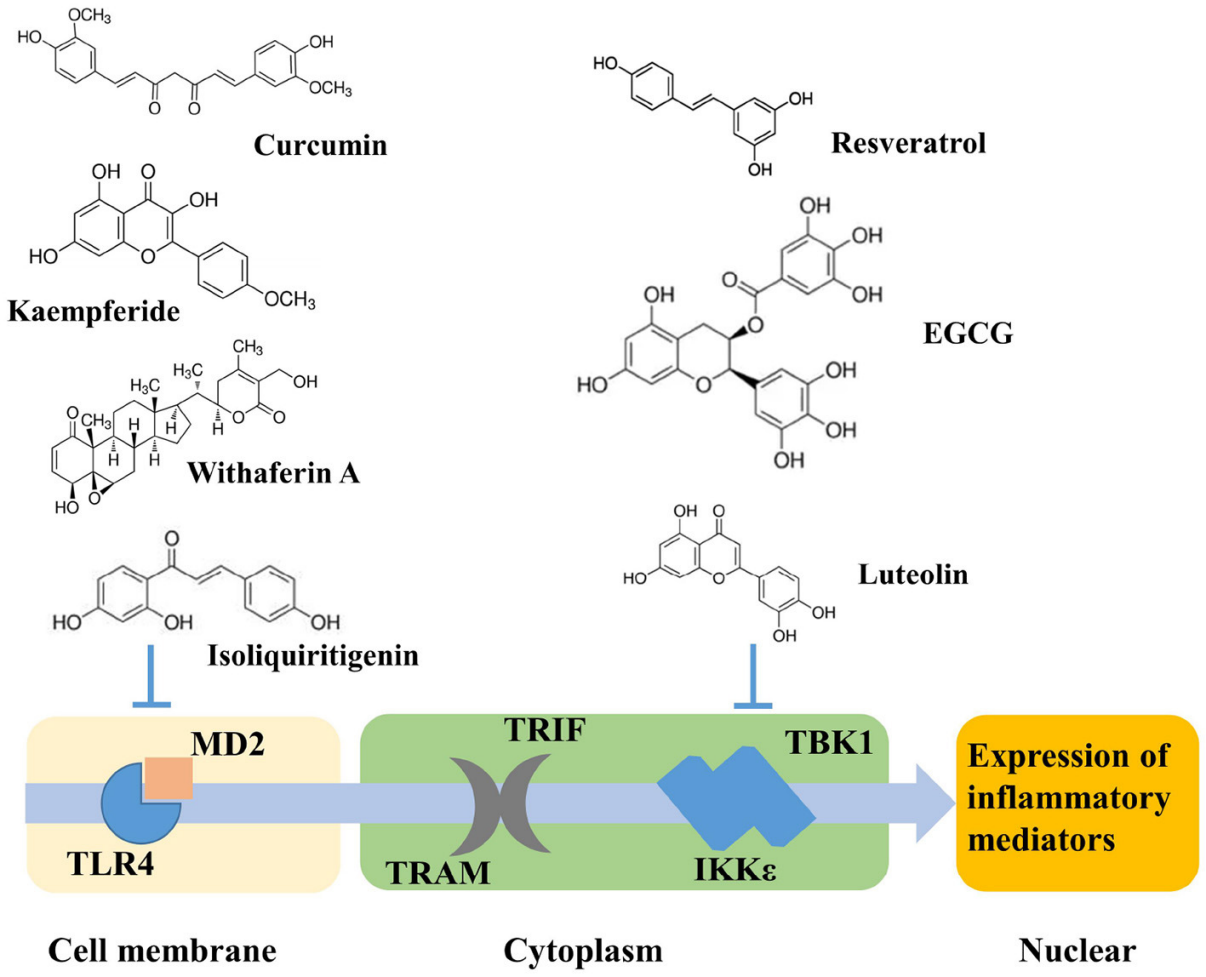
Potential TLR4 signaling pathway induced by phytochemicals.

Cells of myeloid origin such as monocytes and macrophages exhibit the highest levels of TLR4 expression (17). In liver, TLR4 is expressed by hepatocytes and non-parenchymal cells (NPCs), including liver sinusoidal endothelial cells (LSECs) and Kupffer cells (KCs) (18). Stimulation of TLR4 by ligands such as lipopolysaccharides (LPS), leads to the activation of two downstream signaling pathways: MyD88-dependent and MyD88-independent (TRIF-dependent) signaling pathways. Pro-inflammatory cytokines, such as tumor necrosis factor alpha (TNFα), are typically the major final product of the TLR signaling pathway (Figure 1). TNFα plays an important role in lipid metabolism as well as hepatocyte cell death in the development of obesity (19–23), which promotes lipid accumulation in hepatocytes induces insulin resistance, increases FFA levels, and sustains intracellular lipid retention (19). On the other hand, TNFα promotes hepatic cholesterol accumulation by inducing expression of LDL receptor and by inhibiting efflux of cholesterol (21). TLR4 knockout mice have been reported to protect against insulin resistance induced by high fat diet (HFD) (8). In obese individuals, elevated expression of TLR4 and the adaptor proteins including MyD88, interleukin-1 receptor-associated kinase 1 (IRAK1) and factor receptor-associated factor 6 (TRAF6), was observed to correlate with the elevated expression of TNF-α and interleukin-6 (IL-6) (24, 25).

**Figure 2:**
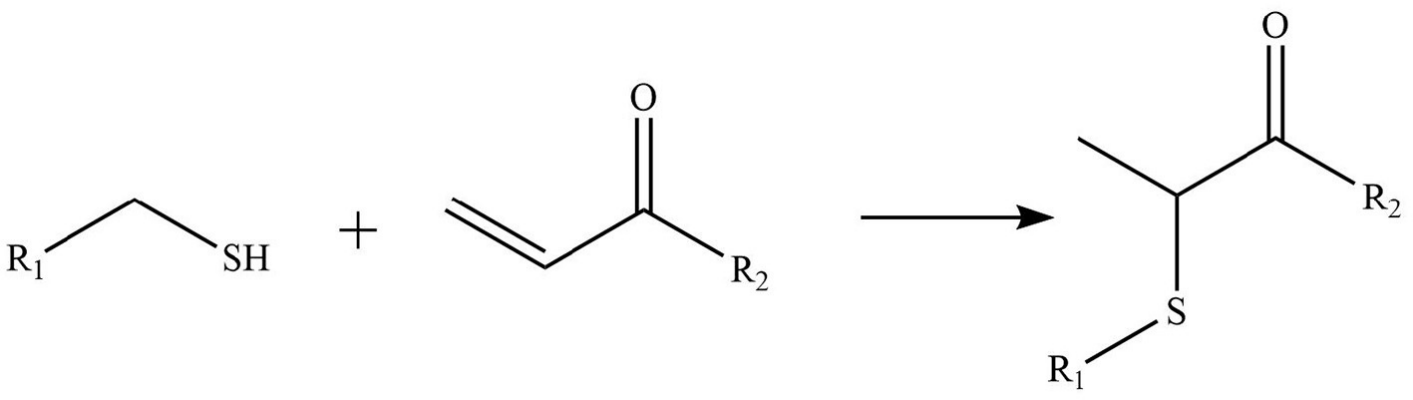
Reaction of thiol groups in the TLR4 signaling pathway with the α,β-unsaturated carbonyl group of phytochemicals.

Phytochemicals that inhibit the activation of TLR4, may ameliorate obesity associated symptoms. It has been well-documented that molecules with the α, β-unsaturated carbonyl groups can react with biological nucleophiles such as a sulfhydryl group (thiol group) by a Michael addition (Figure 2) (26–28). Phytochemicals with α, β-unsaturated carbonyl groups including withaferin A, kaempferide, isoliquiritigenin and curcumin were reported to ameliorate obesity and related metabolic symptoms by the suppression of TLR4.

Withaferin A, extracted from *Withania somnifera* plant, has been verified to attenuate several metabolic diseases. Mohamad reported that administrate with withaferin A (1.25 mg/kg/d) for 12 weeks protected against high-fat diet induced obesity through reducing hepatic mRNA expressions of TLR4, NF-κB, TNF-α), chemokine (C-C motif) ligand-receptor, and cyclooxygenase 2 (COX2) (29).

Tang and colleagues found that obesity, glycolipid metabolism disorder, inflammation, and oxidative stress were effectively alleviated by kaempferide treatment for 16 weeks in HFD mice, and the beneficial effects of kaempferide may be associated with inhibition of the TLR4/IκBα/NF-κB signaling pathways (30).

Isoliquiritigenin is a flavonoid derived from *Glycyrrhiza uralensis*, which was proved to improve HFD-induced adipose tissue fibrosis with decreased expression of TLR4 (31).

The diarylheptanoid curcumin is a naturally occurring yellow pigment found in the plant curcuma longa. It has been reported that curcumin exhibited anti-inflammatory property by interference with TLR4 and its downstream signaling pathway in HFD-induced obese mice (32–34). The molecular interactions between curcumin and TLR4 including: (1) Curcumin is also known to inhibit the activation of IkB kinase β (IKKβ), which is the main downstream of TLR4. The α, β-unsaturated carbonyl group of curcumin reacts with thiol group containing cysteine residue in the activation loop of IKKβ (35). (2) TLR4 dimerization is blocked by curcumin: the α, β-unsaturated carbonyl group of curcumin interacts with free thiol groups in cysteine residues in extracellular and cytoplasmic domains of TLR4. Therefore, curcumin can inhibit LPS-induced activation of both MyD88- and TRIF-dependent pathways of TLR4, and results in the inhibition of both NF-kB and interferon regulatory factor 3 (IRF3) (36).

On the other hand, phytochemicals such as resveratrol, epigallocatechin-3-gallate (EGCG), quercetin, luteolin, and analogs of luteolin, do not inhibit TLR4 dimerization. Instead, they inhibit TLR4 signaling by specifically inhibiting TANK binding kinase 1 (TBK1) kinase activity and consequently downregulate the expression of TBK1-targeted genes, including TNF-α, and IL-6 (37). Nevertheless, a clear understanding of the molecular determinants of TBK1 regulation and substrate selection has not been achieved. TBK1 activity can be regulated by phosphorylation of the serine 172 residue within the kinase activation loop (38, 39). Future work is warranted to investigate how specifically these phytochemicals regulated TBK1 kinase activity.

## Peroxisome Proliferator-Activated Receptors (PPARs) Regulation

**Figure 3:**
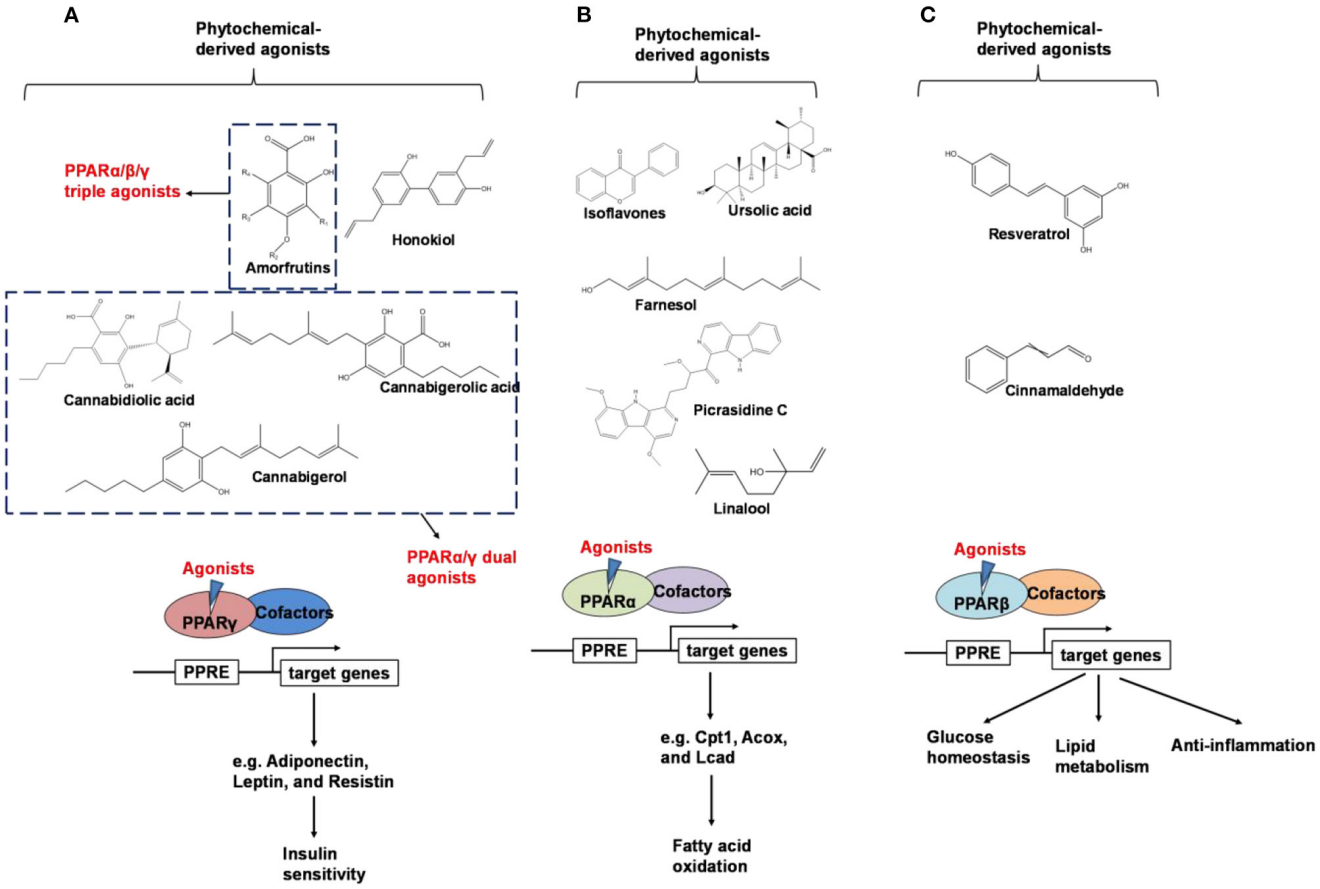
An overview of phytochemical-derived agonists and the biological function of PPARs. **(A)** Agonists of PPARγ. **(B)** Agonists of PPARα. **(C)** Agonists of PPARβ.

PPARs belong to a subfamily of the nuclear receptor superfamily of ligand-inducible transcription factors (40). Three PPAR subtypes including PPARα, PPARβ (also known as PPARδ), and PPARγ, have been identified (41, 42). Peroxisome proliferator-activated receptors control the expression of genes involved in adipogenesis, lipid metabolism, inflammation, and the maintenance of metabolic homeostasis, as summarized in Figure 3 (43).

The regulation of gene transcription is identical in all three PPAR subtypes. Upon ligand binding, PPARs form heterodimers with retinoid X receptor (RXR). The PPAR-RXR heterodimer translocate to the nucleus, where it binds to peroxisome proliferator response elements (PPREs) in the promoter region of the target genes. The transcription process is then initiated, since a conformational change in PPAR-RXR complexes cause the dissociation of co-repressors and recruitment of transcriptional cofactors, while in the absence of a bound ligand, the heterodimer remains bound to the promoter region of its target genes in complex with co-repressors and associated histone deacetylases and chromatin-modifying enzymes, inhibiting the activation of target genes (44, 45).

PPARγ is predominantly expressed in the adipose tissue and plays a central role in lipid and glucose metabolism (46). PPARγ is activated by binding with small lipophilic ligands, mainly fatty acids. Synthetic PPARγ agonists, such as thiazolidinediones (TZDs), which were first reported as insulin-sensitizing drugs in the early 1980s (47), and have been widely used as a therapeutic compound in the treatment of type 2 diabetes, but their underlying mechanism remain unclear until the middle 1990s, when scientists found TDZs were ligands for PPARγ (48).TZDs-induced activation of PPARγ regulates the production and secretion of adipokines, including adiponectin, leptin, and resistin, which impact insulin sensitivity through endocrine signaling pathways (49–51).

Aside from the availability of agonists, the transcriptional activity of PPARγ is also regulated by its phosphorylation status (52). The ability of ligand to suppress Ser273 phosphorylation is well-correlated with their anti-diabetic effectiveness. TZDs inhibit the Cdk5-mediated Ser273 phosphorylation of PPARγ in adipose tissue (52), which in turn, down-regulate expression of genes involved in obesity, including adipsin, a fat-cell-selective gene, and adiponectin, an insulin-sensitizing adipokine (53). PPARγ ligands with poor agonistic activity but potent anti-diabetic effects were revealed to be strong inhibitors of the PPARγ phosphorylation by Cdk5 (54). Consequently, suppression of Ser273 phosphorylation of PPARγ was suggested as a promising approach for development of a new generation of anti-diabetic agents (53).

Activation of another set of genes leads to side effects of PPARγ activation. For instance, TZDs induce the expression of genes involved in adipocyte differentiation and fatty acid storage, such as adipocyte fatty acid binding protein (aP2) (55), phosphoenolpyruvate carboxykinase (PEPCK) (56), and lipoprotein lipase (LPL) (57). It has been reported that ectopic expression of PPARγ in non-adipogenic cells converts them into adipocytes (55), whereas PPARγ knockout mice are unable to develop adipose tissue (58). TZDs promote fat accumulation in type 2 diabetic patients is an example of these undesirable clinical side effects (59).

TZDs was fallen into disuse for their side effects and adverse events in recent years. Water retention was one of the serious clinical events due to TZDs, as well as edema and heart failure (60). The use of TDZs in diabetes clinic visits fell sharply as the side effects reported repeatedly, from 41% in 2005 to 16% in 2012 (61). Therefore, the use of selective PPARγ modulators is a potential way to prevent the side effects of some PPARγ agonists. Different binding modes influence the recruitment of coactivators and elicit a lower transactivation potential of the receptor, which results in the maintenance of antidiabetic activity while minimizing potential side effects of PPARγ modulators (62). The PPARγ ligand binding domain (LBD) consists of 13 α-helices, H1–H12 and H2', and one β-sheet region. Full agonists stabilize H12 by forming hydrogen bonds with the side chains of Ser 289, His 323, His 449, and Tyr 473. However, selective PPARγ modulators stabilize the β-sheet through a hydrogen bond with Ser 342 and helix H3 by hydrophobic interactions (63, 64), while other selective PPARγ modulators also display weaker interactions with the residues that stabilize H12 (65).

Therefore, inhibition of phosphorylation of Ser 273 in PPARγ, as well as the use of selective PPARγ modulators are two preferred therapeutic strategies for improving insulin sensitivity while preventing adipogenesis (53, 66).

Weidner et al. (66) found that amorfrutin, a family of isoprenoid-substituted benzoic acid derivatives from edible parts of Glycyrrhiza foetida and Amorpha fruticosa, possesses powerful antidiabetic effect. In diet-induced obese and db/db mice, amorfrutin treatment strongly improved insulin resistance, decreased plasma triglycerides and inflammatory parameters without concomitant increase of lipid accumulation or other unwanted side effects such as hepatoxicity (66). It might because that amorfrutins bind to and modulate PPARγ, which results in selective gene expression and physiological profiles markedly different from activation by synthetic TZDs. On the other hand, amorfrutins block HFD-induced PPARγ Ser273 phosphorylation in mouse adipocytes, leading to dysregulation of a large number of genes whose expression is altered in obesity (52, 66). Other natural products, such as honokiol, a lignan isolated from the bark, seed cones, and leaves of trees belonging to the genus Magnolia was also reported to improve metabolic parameters in diabetic animal models, with reduced side effects in comparison to TZDs agonists (54, 67).

PPARα is widely expressed in tissues with high fatty acid catabolic activity, including adipose tissue, heart, muscle, liver, kidney, and intestine (68). PPARα is also found to be widely expressed in the digestive tract and hippocampus (69). Activation of PPARα improves insulin resistance, promotes fatty acid catabolism, and inhibits transcription of genes related to the inflammatory response (70).

Several endogenous PPARα ligands have been proposed, including phospholipid 1-palmitoyl-2-oleoyl-sn-glycerol-3-phosphocholine, fatty acids such as palmitic acid, oleic acid, linoleic acid, arachidonic acid, oleoylethanolamide, 3-hydroxy-(2,2)-dimethyl butyrate, hexadecanamide, and 9-octadecenamide (69, 71, 72). Synthetic PPARα agonists such as fibrates lower triglyceride levels and raise high density lipoprotein (HDL), and are used to treat severe hypertriglyceridemia (73). PPARα knockout mice display a fatty liver phenotype (74, 75). Overexpression of PPARα improves glucose tolerance in diet-induced obese mice (76).

Shay and Banz (77) suggested the effect of soy intake on lipid metabolism may be due to isoflavones, the major bioactive compound in soy, acting as activator of PPARα. Linalool, aroma and flavors in most herbal essential oils and teas, was found to act as a direct ligand of PPARα and reduce plasma TG levels, and the reduction was markedly attenuated by silencing PPARα expression (43). Ursolic acid, a natural triterpene compound found in various fruits and vegetables, is a PPARα agonist and was found to control the expression of genes related to lipid metabolism (78, 79). Goto et al. (78) reported that farnesol, a natural organic compound which is an acyclic sesquiterpene alcohol, mediated improvement of obesity-associated metabolic disorders through a PPARα-dependent manner. Picrasidine C, a dimeric β-carboline-type alkaloid isolated from the root of *Picrasma quassioides*, was identified as a selective PPARα agonist by binding with PPARα LBD forming hydrogen bonds with Cys276 and Thr279, therefore exhibiting potential in treating hyperlipidemia, atherosclerosis, and hypercholesterolemia (80).

As reviewed by Rigano et al., structurally related compounds may have robust differences on binding affinity to PPARα. For instance, daidzein or formononetin slightly activated PPARα, while the metabolite 6-hydroxydaidzein exerted a much higher agonistic PPARα activity. Similarly, 3′-hydroxygenistein exhibited a more potent on PPARα activation than its precursor genistein. Biochanin A, differing from genistein only by methylation of the 4'OH group, was robustly more potent than its precursor. In contrast, the metabolites dihydrogenistein and dihydrodaidzein did not transactivate PPARα (69, 81). The pentacyclic triterpene oleanolic acid was found to stimulate PPARα activation in keratinocytes while the closely related ursolic acid, differing only by the methylation pattern on ring E, failed to exhibit this activity (82). The reason for the differences is not well-understood: it may be related to an increase in the bioavailability of the molecules, an improved interaction with the binding site, different abilities to recruit coactivators or co-repressors, and/or cross-activation of other nuclear receptors (69).

As a phosphoprotein, PPARα can also be activated through post-translational modification. The phosphorylation sites in PPARα include Ser12, Ser21, Ser179, and Ser230 (83–87). The increased transactivation of PPARα may occur via decreased co-repressor interaction, such as NCoR, or increased interaction with co-activators, such as Peroxisome proliferator-activated receptor gamma coactivator 1-alpha (PGC-1α) (88). Since PGC-1α is one of the major transcription factors of non-shivering thermogenesis (89), phytochemicals that potential to activate its co-activator PPARα draws a lot of attention to induce thermogenesis in rodent models. Li et al. (90) reported that tyrosol, one of the main polyphenolic compounds in extra virgin olive oil, acted as a ligand which binds with PPARα, and increased its downstream genes expression, such as uncoupling protein 1, iodothyronine deiodinase 2 and PGC-1α, resulted in reduced body weight gain in HFD-induced obese mice.

Peroxisome proliferator-activated receptor β (PPARβ) is expressed ubiquitously (91). PPARβ, also a lipid ligand-inducible transcription factor, regulates lipid metabolism and glucose homeostasis. PPARβ is also shown to suppress the activities of several transcription factors, including NFκB, and activator protein 1, thus regulating anti-inflammatory cellular responses (92, 93). Compared to non-obese subjects, obese patients exhibited reduced PPARβ expression in both the subcutaneous and visceral adipose tissues (94). Previous reports have shown that PPARβ agonists, such as GW501516, ameliorates insulin resistance by increasing the expression of the insulin receptor, of significance for treatment of insulin resistance in patients with type 2 diabetes mellitus (95). Consistently, administered with the PPARβ agonist MBX-8025 for 8 weeks, overweight patients presented favorable trends in the body fat percentage, lean body mass and waist circumference (96).

Resveratrol is a natural stilbene found in grapes and red wine. Tsukamoto et al. (97) demonstrated that resveratrol treatment activated PPARβ in bovine arterial endothelial cells. Qin et al. (98) showed that resveratrol was able to regulate PPARβ expression in retinal pigment epithelial cells in a dose-dependent manner. In addition, Lu et al. suggest that the treatment of resveratrol, modulated adipokine expression and improves insulin sensitivity in adipocytes by down-regulating PPARβ (99).

Since the ligand binding domains among PPARs are 60–70% identical (100), phytochemicals may act as dual agonists for PPARs or as pan agonists. For example, D'Aniello et al. identified cannabigerolic acid, cannabidiolic acid and cannabigerol from C. sativa as PPARα/γ dual agonists (101). Amorfrutin has binding affinities for PPARα, PPARβ, and PPARγ (66).

## Regulation of Keap1/Nrf2/ARE Signaling Pathway

Nuclear factor erythroid 2-related factor 2 (Nrf2), a transcription factor in the basic leucine zipper (bZIP) superfamily, is an attractive target for obesity and metabolic syndrome treatment and prevention. Dual roles of Nrf2 in obesity and related metabolic diseases, including the regulation of antioxidant defenses and hepatic fatty acid metabolism have been reported (102, 103).

Nrf2 behaves as a cellular redox status sensor and under normal circumstances, most Nrf2 is sequestered, bound to the cytoskeletal-anchoring protein Kelch-like ECH-associated protein 1 (Keap1), a cysteine rich, homodimeric zinc-finger protein in the cytosol. Keap1 acts as a substrate adaptor protein for the cullin 3-containing E3-ligase complex and targets Nrf2 for ubiquitination, which leads to proteasome mediated degradation of Nrf2. During the exposure of electrophilic or oxidative stressors, Nrf2 releases from sequestration as stressors interact with cysteine thiols of Keap1, followed by translocation to the nucleus. Nrf2 dimerizes with small Maf proteins or other leucine zipper proteins, binds to antioxidant response elements (ARE) in the promoter or enhancer of target genes, and thus regulates genes involved in the protection against oxidative/electrophilic stress (104, 105). These target genes include NAD(P)H dehydrogenase, quinone 1(*Nqo1*), glutathione S-transferase (*Gst*), and heme oxygenase-1 (*Ho-1*) (103). Electrophilic phytochemicals, such as curcumin and sulforaphane, possess α,β-unsaturated carbonyl groups which interact with thiol groups in cysteine residues of the Keap1 protein, releasing Nrf2 into the nucleus, thus activating downstream genes (106).

**Figure 4:**
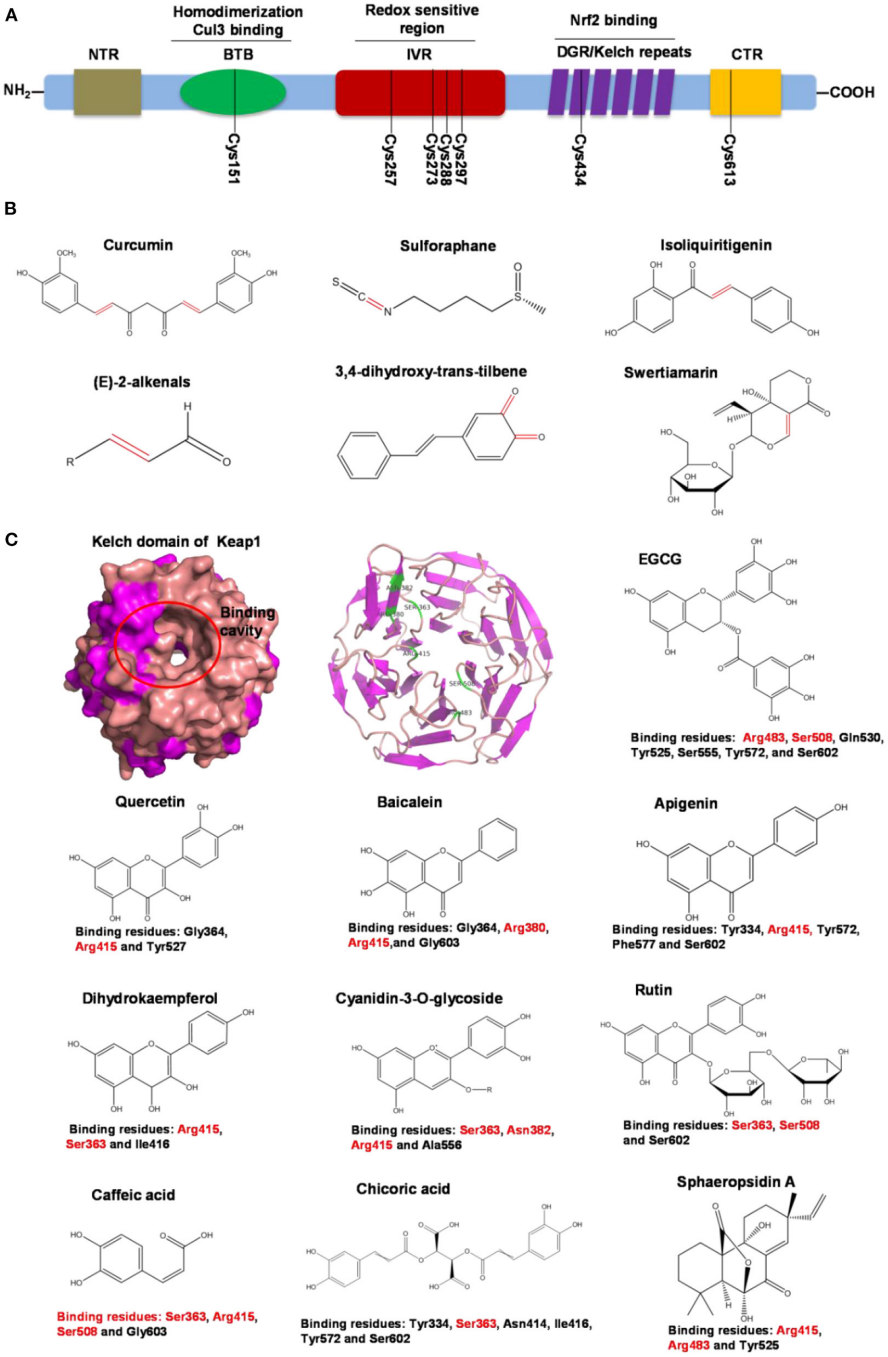
Summary of Keap1 protein structure and binding to phytochemicals. **(A)** Domain structures of Keap1 protein. Seven cysteine residues (Cys151, Cys257, Cys273, Cys288, Cys297, Cys434, and Cys613) are responsible for covalent binding with phytochemicals to activate Nrf2. **(B)** Phytochemicals with α, β-unsaturated carbonyl groups having highly potent binding with cysteine residues of Keap1 through covalent modification. **(C)** Non-covalent binding cavity and residues of Kelch domain of Keap1 with phytochemicals. The reactive unsaturated carbonyl group of phytochemicals and potential non-covalent binding residues of Kelch domain of Keap1 are highlighted using red font.

Human Keap1 has at least 25 reactive thiols, most of which are found in the IVR (intervening linker region) redox-sensitive region (107, 108). Specially, seven critical cysteine residues, including Cys151, Cys257, Cys273, Cys288, Cys297, Cys434, and Cys613 (Figure 4A), are responsible for sensing of alkenals and redox signals, which are required for the stimulation of Nrf2 (109). Not all phytochemicals preferably interact with the same region of Keap1 protein. Luo et al. found that for isoliquiritigenin, the top reactive thiols on human Keap1 were cysteine residues Cys151 and Cys266. However, for xanthohumol (a prenylated flavonoid isolated from hops) and 10-shogaol (pungent constituents of ginger), top reactive thiols on Keap1 were Cys151, Cys319, Cys613 and Cys151, Cys257, Cys368, respectively (110). The different interactions between these electrophiles with Keap1 might be due to their unique structures and reactivities (Figure 4B). It is imperative to investigate the reason for these differences.

Notably, electrophilic Nrf2 activators might be not highly selective for Keap1 over other cytoplasmic ubiquitous cysteines due to their action involving the covalent reaction with cysteine thiols, which leads to non-specific effects by perturbing multiple targets except for Keap1 (111). Alternatively, direct and non-covalent disrupting of Keap1-Nrf2 protein-protein interaction (PPI) has emerged as a rationale for selective activation of Nrf2. The C-terminal Kelch domain of Keap1 is responsible for its binding with Nrf2 (109). Among Kelch domain residues, three highly conserved arginine residues (Arg380, Arg415, and Arg483) and other serine, glutamate and asparagine residues (Ser363, Ser508, and Asn382) play important roles in Keap1 binding to Nrf2 (112). Numerous common natural phytochemicals (quercetin, EGCG, baicalein, caffeic acid, sphaeropsidin A, dihydrokaempferol, rutin, apigenin, and anthocyanins) can bind to Kelch domain through the formation of non-covalent bonds (H-bond, π-π/H-π interaction, hydrophobic effects, van der Waals and ionic bonds) with the specific residues involving Tyr334, Ser363, Gly364, Asn382, Arg415, Arg483, Ser508, Tyr525, Tyr527, Gln530, Ser555, Ala556, Tyr572, Phe577, Ser602, and Gly603, thereby directly disrupting Keap1-Nrf2 interaction to activate Nrf2 (Figure 4C) (106, 113–119). However, these interactions of the Kelch domain of Nrf2 with phytochemicals were predicted based on molecular docking, which should be further compared and verified by using direct biomolecular interaction techniques, such as isothermal titration calorimetry, surface plasmon resonance, and biolayer interferometry.

Besides activating antioxidant genes, Nrf2 is also known to participate in the regulation of hepatic fatty acid metabolism in rodents, as a negative regulator of genes that promote hepatosteatosis (120). Nrf2 has been reported to directly impact the regulation of genes including ATP citrate lyase (*Acly*), acetyl-CoA carboxylase 1 (*Acaca*), fatty acid synthase (*Fasn*) and fatty acid elongase 6 (*Elovl6*) (104, 121). Hepatic lipogenesis is negatively regulated by Nrf2 in mice administrated a high fat diet (122, 123).

Activation of Nrf2 pathway *via* phytochemical supplementation has been found to protect mice from obesity. In a study by Okada et al. (124) sulforaphane supplementation suppressed oxidative stress and hepatic fibrosis in mice induced by MCD diet. Sulforaphane also activates lipolysis by transcriptionally regulating genes related to lipid metabolism, including adipose triglyceride lipase (*Atgl*) and hormone-sensitive lipase (*Hsl*) (125). Nagata et al. (126), revealed the anti-obesity effect of glucoraphanin, precursor of sulforaphane, in HFD-fed mice, and found that 0.3% w/w glucoraphanin for oral administration for 14 weeks significantly reduced body weight, alleviated hepatic steatosis and improved insulin sensitivity in wild type mice but not in Nrf2 KO mice. They further investigated that whole body energy expenditure was increased, accompanied with over expression of *Ucp1* in WAT, but these results were not found in Nrf2 KO mice. Thus, stimulation of energy metabolism by Nfr2 activation was considered effective to combat obesity. Other natural phytochemicals (i.e., sesamol, curcumin, *Garcina cambogia*, timosaponin) that can activate Nrf2 are potential candidates to prevent obesity and improve metabolic disease *via* Nrf2 pathway (127–130).

Interestingly, mice deficient in Nrf2 were protected from high fat diet induced obesity, including improved glucose tolerance, reduced hepatic triglyceride content and decreased liver weight (131, 132). Chartoumpekis et al. (133) found that Nrf2 knockout mice displayed decreased fat mass in association with small adipocytes and are resistant to diet-induced obesity. Xu et al. (134) found that enhanced Nrf2 activity induced insulin resistance in leptin-deficient mice. The mechanisms proposed include interaction of Nrf2 with other pathways (Fibroblast Growth Factor 21, or lipid synthesis enzymes) mainly in liver and white adipose tissue (135).

Despite contrary findings that both Nrf2 gain or loss of function may protect from obesity, this likely happens through distinct mechanisms/pathways. A detailed assessment of obesity in mouse models with Nrf2 deletion or overexpression is warranted to determine the molecular pathways underlying the positive and negative effect of Nrf2.

## Regulation of Fat Mass and Obesity-Associated Protein (FTO) Signaling Pathway

**Figure 5:**
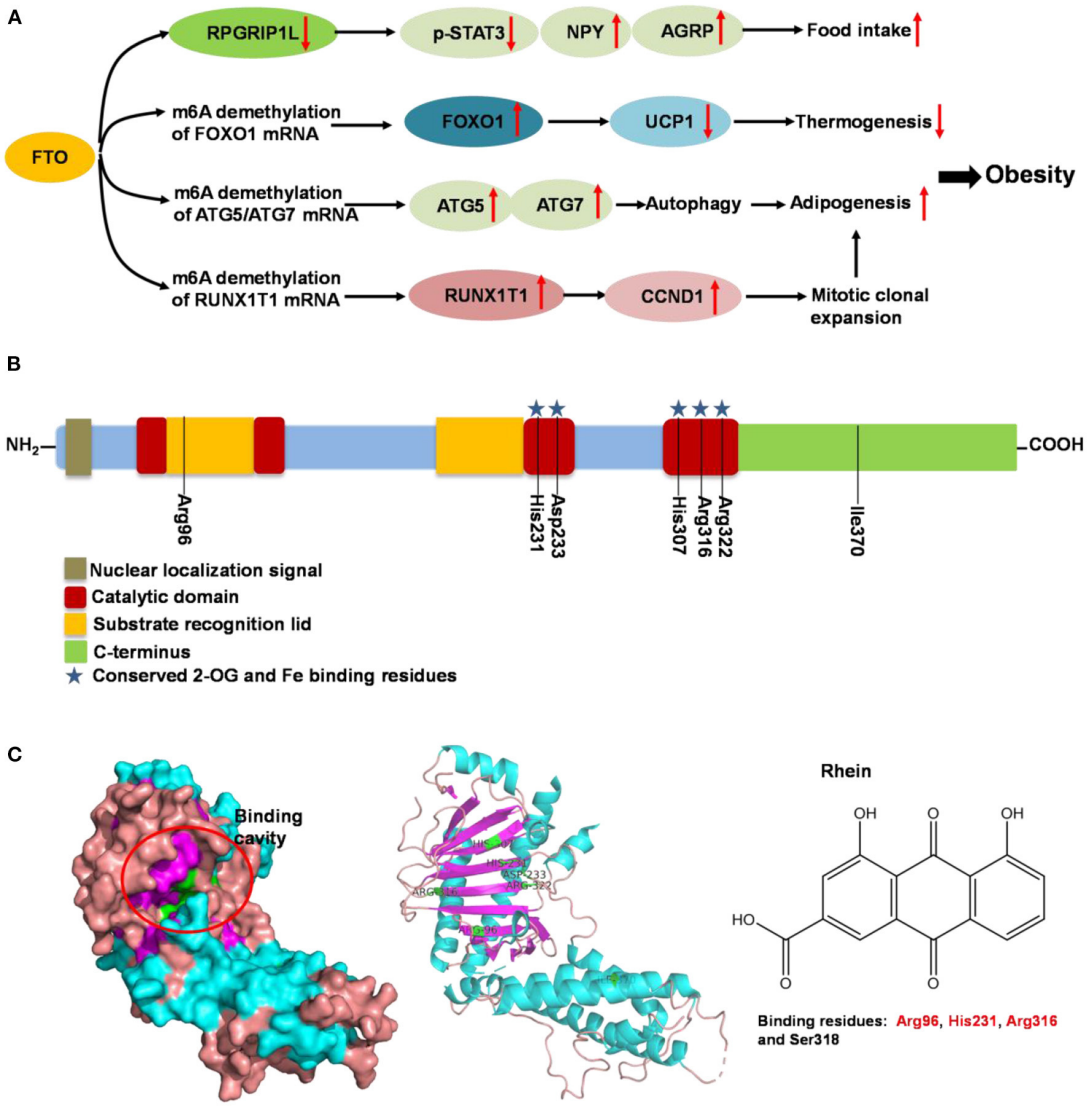
Summary of molecular mechanism of FTO-induced obesity, FTO protein structure and its binding with rhein. **(A)** Mechanism of FTO-induced obesity through regulating food intake, thermogenesis and adipogenesis. **(B)** Domain structures of FTO protein. Seven amino acid residues (Arg96, His231, Asp233, His307, Arg316, Arg322, and Ile370) are responsible for FTO activity. **(C)** Binding cavity and residues of FTO protein with rhein. The potential binding residues of FTO are highlighted using red font.

Fat mass and obesity-associated protein (FTO) is a member of the Fe (II)- and oxoglutarate-dependent AlkB dioxygenase family, and is known for the strong association of the multiple single-nucleotide polymorphisms located in its intron 1 with risk of obesity (136). FTO knockout or loss-of-function mutations lead to reduced body weight, and its overexpression contributes to obesity (137). The mechanism of FTO-induced obesity is summarized in Figure 5A: FTO expression in the brain positively regulates food intake through neuropeptide Y (NPY) and ghrelin (137, 138). FTO in the adipose tissue has been reported to control the expression of uncoupling protein 1, a mitochondrial inner membrane proton channel linked to thermogenesis, through FOXO1 (139). Adipogenesis is promoted by FTO by targeting Atg5- and Atg7-mediated autophagy as well as adipogenic regulator factor Runx1t1 (140, 141). The role of FTO in controlling thermogenesis and adipogenesis in the adipose tissue largely depends on its regulation of m6A mRNA methylation, which is the most abundant mRNA modification in mammals and is involved in various biological processes including obesity and obesity associated-metabolic disorders (139, 142).

Following the determination of the FTO crystal structure by Chai's group in 2010 (Figure 5B) (143), FTO is widely viewed as an attractive biological target; potentially a small-molecule inhibitor specifically targeting FTO could be developed for the treatment of metabolic disorders such as obesity and diabetes. Some synthetic compounds, such as CHTB, FB23, and diacerein, occupying the αKG and/or substrate binding site have been identified as potent inhibitors of FTO, potentially regulating obesity (144, 145). Peng et al. (139) used a structure-based hierarchical virtual screening approach to identify potential FTO inhibitors from 1,323 FDA-approved drugs, and found entacapone as a chemical inhibitor of FTO which directly binds to FTO and inhibits its demethylation activity, mediating metabolic regulation through FOXO1. Development of FTO-specific inhibitors from natural phytochemicals is a promising strategy to avoid the side effects of synthetic drugs for treatment of obesity and other chronic diseases (146). The natural phytochemical Rhein was identified as the first FTO inhibitor that disrupts FTO activity by directly binding to the catalytic domain, which blocks access to the ssRNA substrate (Figure 5C) (147), and rhein remarkably suppresses adipogenesis in the stage-specific and dose-dependent manners (https://pubmed.ncbi.nlm.nih.gov/34790688/). But, side-by-side comparison analysis revealed that the rhein treatment and FTO knockdown triggered the differential gene regulatory patterns, though both resulting in impaired adipocyte formation, suggesting separate regulation of global m6A pattern and adipogenesis mediated by rhein (148). So, the interrelation of rhein-FTO- adipogenesis remain elusive. Epigallocatechin gallate has been shown to inhibit adipogenesis by regulating FTO expression in an mRNA m6A-dependent manner (149). Baicalin has been reported to ameliorate high-fat diet-induced obesity and hepatic steatosis through carnitine palmitoyltransferase 1 (CPT1) (150), of which mRNA serves as a potential substrate of FTO for m6A modification, predicted by m6A-Atlas (151). Zhong et al. (152, 153), reported that *Angelica sinensis* alleviated HFD-induce obesity through altering expression of FTO gene. Betaine was also found to decrease FTO expression and improved m6A methylation in adipose tissue of wild-type mice with high-fat diet, resulting in decreased final body weight and improved glucose tolerance (154). In another human trial included 214 participants revealed that, Garcinia cambogia supplement with 1,000 mg/day for 6 months significantly reduced body weight and improved serum lipid profile, but these beneficial effects were hampered by polymorphisms of FTO gene (155). Suggesting that, FTO may at least partially involved in this anti-obesity process.

## MicroRNA (miRNAs) Pathway Regulation

The miRNAs are a class of regulatory RNAs. They are small non-coding RNA molecules, averaging 22 ribonucleotides in length, and may repress gene expression post-transcriptionally by binding to untranslated regions and coding sequences of target mRNAs (156). The class of RNA was first discovered in *Caenorhabditis elegans* in 1993, and then identified in vertebrates and plants (157).

The miRNA coding sequences are transcribed by RNA polymerase II to yield primary miRNAs (pri-miRNAs) in the nucleus, and further processed by Drosha RNAse III endonuclease and microprocessor complex subunit DGCR8 to release precursor miRNAs (pre-miRNAs), which are about 60–70 ribonucleotides long. The pre-miRNAs are transported into the cytoplasm by Exportin-5 and cleaved by Dicer complex, another RNase III enzyme, to generate miRNA/miRNA double stranded molecules (158). Upon separation of the two strands, the guide strand binds to an Argonaute (Ago) protein and is integrated into the RNA-induced silencing complex (RISC), where it targets and binds to the 3′-untranslated region (3′UTR) of target mRNAs *via* base pair complementarity (159). This binding leads to degradation or translational repression of target mRNAs (160).

Thousands of different miRNAs have been identified, and miRNAs are now recognized as one of the most abundant classes of gene-regulatory molecules in multicellular organisms (161). Computational and experimental studies have shown that some miRNAs play a role in lipid metabolism and glucose homeostasis, therefore, may be influencing the pathogenesis of metabolic diseases (162).

MicroRNA-122 (miR-122), an abundant liver-specific miRNA, accounts for approximately 70% of total miRNAs in the liver. microRNA-122 is the first miRNA to be linked recently to fat and cholesterol metabolism, suggesting it as a therapeutic target for metabolic diseases (163, 164).

Temporary miR-122 inhibition resulted in reduced plasma cholesterol levels in both normal and diet-induced obese mice (163, 165), by down-regulating hepatic gene expression including HMG-CoA reductase and phosphomevalonate kinase (PMVK), which are involved in cholesterol biosynthesis (163). Consistently, a study of non-human primates demonstrated that miR-122 inhibition caused a dose-dependent decrease in plasma cholesterol, indicating a therapeutic potential in the treatment of hypercholesterolemia in humans (166). A study from Wang et al. found that in young adults, elevated circulating miR-122 is positively associated with obesity and insulin resistance (167).

Excessive retention of triglyceride within hepatocytes was observed in miR-122-knockout mice. Hsu et al. found that both liver-specific and germline miR-122 knockout mice exhibited increased hepatic triglyceride accumulation and progressive steatohepatitis, which was not seen in the mice with temporary miR-122 inhibition (168). The underlying mechanism may be due to the up-regulation of triglyceride biosynthesis related gene expression in the liver: 1-acylglycerol-3-phosphate O-acyltransferase 1 (*Agpat1*) and monoacylglycerol O-acyltransferase 1 (*Mogat1*), (168) and the reduction of microsomal TG transfer protein (*Mttp*) (169), which normally functions to enhance the rate of lipid transfer between vesicles (170).

Phytochemicals which modulate the expression of miR-122 possess the potential to regulate lipid metabolism associated with obesity and metabolic syndrome. Epigallocatechin-3-gallate was found to decrease miR-122 expression. Using ^1^H NMR spectroscopy, EGCG was observed to bind directly to miR-122 through an interaction with all of the rings of the EGCG molecule (171). However, resveratrol was found to bind directly to miR-122 primarily through an A ring interaction, which increases the expression of miR-122. It is hypothesized that the size and chemical structure of these molecules deferentially influenced the expression of miRNA, which may account for the opposite effects of resveratrol and EGCG on miRNA modulation (171).

Other phytochemicals were reported as modulators of miRNA levels. Polyphenols in acai and red muscadine grape protect human umbilical vascular endothelial cells from glucose and lipopolysaccharide-induced inflammation, partly acting through the upregulation of miR-126 expression (172). Zerumbone, a phytochemical isolated from the subtropical Zingiberaceae family, was reported to reverse high-fat diet-induced adiposity by restoring AMPK-regulated lipogenesis and the regulation of miRNA-146b mediated adipogenesis (173). Fisetin, which present in fruits and vegetables such as strawberries, apple, cucumber, persimmon, grape and onion, was shown to suppresses the expression of hepatic miR-378, a miR located in intron of the peroxisome proliferator-activated receptor gammacoactivator-1 beta (PGC-1β), resulting in preventing obesity and hepatic lipid accumulation induced by high fat diet in mice (174). Polyphenols derived from *Hibiscus sabdariffa* were reported to regulate the expression of miR-103, miR-107 and miR-122 and attenuated weight gain, liver steatosis and insulin resistance in hyperlipidemic mice (175).

Emerging data suggest that phytochemicals alter the expression of miRNAs involved in regulation of cancer pathobiology by modulating the expression of miRNAs through mechanisms including epigenetic, transcriptional, and miRNA processing (176). However, relatively little is known about how phytochemicals regulate miRNAs related to obesity and metabolic syndrome (177). It is hypothesized that similar mechanisms might be involved, although more investigation is needed. Since each mammalian miRNA may regulate a large number of target genes, it can be that several different miRNAs can act synergistically at multiple target sites of a single Mrna (178). Thus, it is imperative to manipulate multiple candidate miRNAs in different combinations rather than changing one miRNA at a time for a full functional characterization of their regulatory impact.

## Conclusion

Plenty number of phytochemicals have been reported as candidates for the management of obesity. However, the role of the structure of phytochemicals in specific signaling pathways remains unclear. In this review, we discussed the regulation of phytochemicals on five different signaling pathways involved in obesity and related symptoms (Table 1). In particular, we discussed specific molecular structures required for regulation to occur. We suggest that, in the future, more attention should be paid to the preliminary research on the correlation between structures of phytochemical and specific target molecular using accessible methods, such as molecular docking by computer or *in vitro* kinetic measurements. Although the current evidence is limited, we suggest that in some cases, the optimal molecular structure for a specific pathway to become activated or regulated may ultimately be deduced.

**Table 1:** Phytochemicals targeting specific signaling pathway act against obesity and related symptoms.

**Phytochemicals**	**Target molecular**	**Major results**	**References**
Curcumin 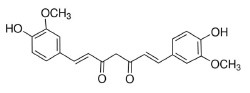	TLR4↓	- Anti-inflammation (↓macrophage infiltration of adipose tissue) and insulin sensitizer (↓serum glucose in GTT) in HFD-mice (0.4% of diet, 14 weeks)	(34)
Kaempferide 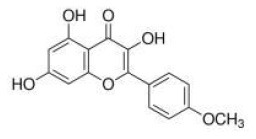	TLR4↓	- Anti-obesity, anti-hyperlipidemia, anti-hyperglycemia, anti-inflammation and anti-oxidation (↓body weight, ↓serum TG, TC, HDL-C, LDL-C, glucose, TNFa, MCP-1; ↑serum adiponectin; ↑liver GSH, SOD, CAT, T-AOC, GSH-Px; ↓liver MDA) in HFD-mice (10 mg/kg bw, p.o., 8 weeks).	(30)
Withaferin A 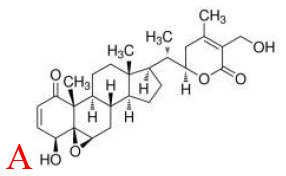	TLR4↓	- Anti-obesity, insulin sensitizer, anti-inflammation and anti-oxidation (↓body weight;↓epididymal WAT weight;↑serum adiponectin, ↓serum leptin, TC, TG, FFA, ALT, AST; ↓serum glucose in GTT and ITT; ↓serum IL-6, TNFa, IL-1b, CRP, MCP-1; ↑liver GSH, SOD, CAT, T-AOC, GSH-Px; ↓liver TBARS) in HFD-mice (1.25 mg/kg bw, p.o., 12 weeks).	(29)
Isoliquiritigenin 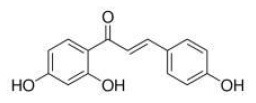	TLR4↓	- Attenuated adipose tissue inflammation in a co-culture model composed of adipocytes and macrophages (↓NF-kB activation, Akt phosphorylation). - Attenuated adipose tissue fibrosis in HFD-induced obese mice (↓ fibrotic area of eWAT).	(31)
Amorfrutin A1 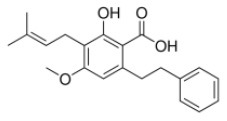	PPARγ–agonist (AG)	- Anti-hyperglycemia and insulin sensitizer (↓body weight; ↓blood glucose; ↓HOMA-IR, ↓blood glucose and insulin in GTT; ↑pancreatic insulin; ↓plasma ALT,TG, FFA) in HFD-obese mice, and ↓ plasma insulin; ↓plasma TG; ↑pancreatic insulin in db/db mice (100 mg/ kg bw, p.o., 3 weeks). - Anti-steatosis (↓body weight; ↓plasma insulin, leptin; ↓liver TG) in HFD-obese mice (37 mg/ kg bw, p.o., 15 weeks).	(66)
Amorfrutin B 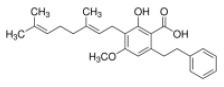	PPARα–AG PPARδ–AG PPARγ–AG	- Anti-hyperglycemia, insulin sensitizer (↓blood glucose, ↓plasma insulin, ↓HOMA-IR index, ↓blood glucose and insulin in GTT; ↓plasma TG and NEFA) in HFD-diabetic mice (100 mg/kg bw, p.o., 27 days).	(179)
Soy isoflavones 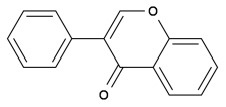	PPARα–AG PPARα–AG	- Anti-hyperlipidemia (↓liver weight, ↓liver cholesterol, ↓liver TG, ↓plasma cholesterol). - Insulin sensitizer (↓plasma insulin (male), ↓serum glucose in GTT) in obese Zucker rats (0.75 g/kg of diet, 11 weeks).	(180)
Linalool 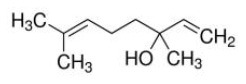	PPARα–AG	- Anti-hyperlipidemia (↓plasma TG) in Western-diet fed C57BL6J and apoE2 mice but not in PPARα -deficient mice (100 mg/kg bw, p.o., 3 weeks).	(43)
Tyrosol 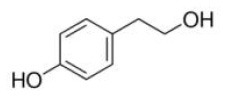	PPARα–AG	- Anti-obesity (↓body weight, ↓liver weight, ↓plasma TG, TC, and glucose) in obese mice (0.2% of diet, 16 weeks).	
Ursolic acid 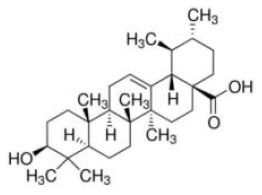	PPARα–AG	- Anti-hyperlipidemia (↓TG and TC content) in HepG2 cells (5–100 mM).	(79)
Picrasidine C 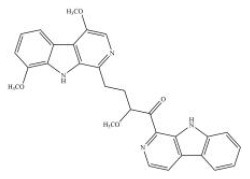	PPARα–AG	- Promoted PPARα transcriptional activity and induced the expression of CPT-1, PPARα, PDK4, and ABCA1 in HepG2 cells (1–20 μM).	(80)
Vaticanol C (resveratrol tetramer)	PPARα and PPARβ/δ–AG	- Activated PPARα and β/δ in bovine arterial endothelial cells (1.25–10 μM). - Upregulated hepatic expression of PPARα-responsive genes and muscle PPARβ/δ-responsive genes in wild-type, but not PPARα-knockout mice in HFD-fed mice (0.04% of diet, 8 weeks).).	(97)
Resveratrol 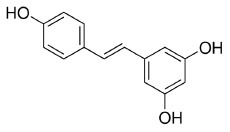	PPARα and PPARδ–AG	- Anti-inflammation (↓ROS, ↓IL-8) in retinal pigment epithelium cells (25 μM).	(98)
Honokiol 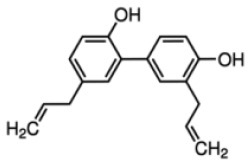	PPARγ–AG	- Induced glucose uptake but not adipogenesis in 3T3-L1 cells (1–10 μM). - Decreased blood glucose levels in diabetic KKAy mice with simultaneous suppression of weight gain.	(181)
Glucoraphanin 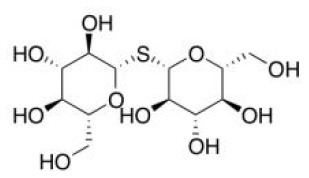	Nrf2↑	- Anti-obesity, anti-hepatic steatosis, insulin sensitizer (↓body weight; ↓fat mass;↑energy expenditure; ↓liver weight, TG, FFA, AST, ALT; ↓serum glucose in GTT and ITT; ↓HOMA-IR index) in HFD-fed wild-type mice but not in HFD-fed Nrf2 knockout mice (0.3% of diet, 14 weeks).	(126)
Sesamol 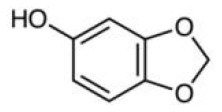	Nrf2↑	- Anti-obesity (↓body weight, eWAT, iWAT; ↓serum ALT, glucose, FFA, LDL-C; ↑energy expenditure) in HFD-mice (100, 200 mg/kg bw, p.o., 12 weeks). - Stimulated expression of UCP1 in wild-type adipocyte but not in Nrf2 knockout cells.	(127)
Curcumin 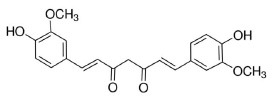	Nrf2↑	- Anti-oxidation (↓serum MDA; ↓mitochondria MDA; ↓muscle MDA, ROS); Insulin sensitizer (↓serum glucose in GTT and ITT; ↓HOMA-IR index) in HFD-mice (50 mg/kg bw, p.o., 18 weeks).	(128)
Garcinia cambogia	Nrf2↑	- Anti-hepatic steatosis, anti-hepatic apoptosis (↓serum ALT, AST, TG, TC) in HFD-mice (200, 400 mg/kg bw, p.o., 8 weeks). - Reduced lipid accumulation, apoptosis, ROS level in FFA-induced HepG2 cells (20–80 ug/ml, 24 h).	(129)
Timosaponin 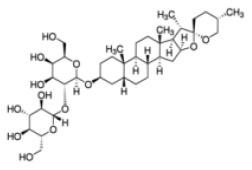	Nrf2↑	- Anti-oxidation (↓serum MDA; ↑serum T-AOC, GSH-Px; ↓liver ROS, MDA; ↑liver GSH-Px; - Anti-obesity (↓body weight, epididymal and retroperitoneal fat weight, number of adipocytes in epididymal and retroperitoneal fat) in HFD-mice (0.1, 0.4 g/kg bw, p.o., 11 weeks).	(130)
EGCG 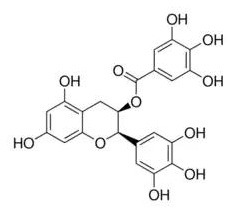	FTO↓	- Anti-adipogenesis (↓adipocyte differentiation, lipid accumulation) in 3T3-L1 cells (50–200 μM, 30 h).	(149)
Clausine E 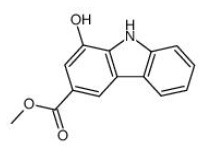	FTO↓	- Inhibition of FTO activity *in-vitro*.	(182)
Garcinia cambogia	FTO	- Anti-obesity (↓body weight, fat mass, visceral fat; ↓serum TC, TG, glucose; ↑basal metabolic rate) in obese humans (214 participants, 1,000 mg/day, 6 months), the presence of polymorphisms FTO might hamper these beneficial effects.	(155)
Angelica sinensis	FTO↑	- Anti-obesity [↓body weight (10 g/kg bw)] and promoted methylation of CpG island in the FTO promoter in HFD-mice (2, 5, 10 g/kg bw, p.o., 4 weeks). - FTO expression in high dose group (10 g/kg bw) were significantly higher than control.	(153)
EGCG 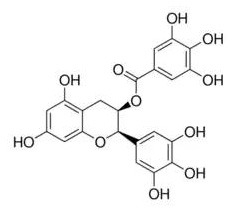	↓miR-33a and miR-122	- Increase ABCA1 mRNA and protein level, not altered FAS mRNA, slightly decreased FAS protein level in HepG2 cells (50 μM).	(171)
Resveratrol 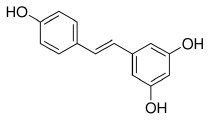	↑miR-33a and miR-122	- Increased ABCA1 protein level, and increased FAS mRNA and protein in HepG2 cells (50 μM).	(171)
Zerumbone 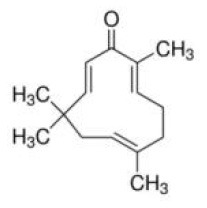	↓miR-146b	- Anti-obesity, insulin sensitizer (↓body weight; ↓eWAT; ↓adipocyte size; ↓serum triglyceride; ↓serum insulin; ↓glucose; ↓HOMA-IR) (0.01%, 0.025% of diet, 8 weeks). - Anti adipogenesis in 3T3-L1 cells (↓C/EBPα, PPARγ, FASN, Ap2) (5, 10 μM for 48 h).	(173)
Fisetin 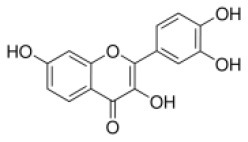	↓miR-378	- Anti-hepatosteatosis (↓body weight; ↓serum cholesterol and triglyceride; ↓fat accumulation and triglyceride levels of liver in HFD-feed mice (0.5% of diet, 10 weeks).	(174)
Polyphenols extracted from Hibiscus sabdariffa	↓miR-103 and miR-107	- Anti-obesity, anti-hepatic steatosis, insulin sensitizer (↓body weight; ↓adipocyte size of eWAT; ↓serum insulin, glucose, HOMA-IR) in HFHC-feed mice.	(175)

*↑, increased; ↓, decreased*.

## Author Contributions

Z-YD and TL: conceptualization. XL: writing—original draft preparation. BZ and LZ: writing—review and editing. All authors contributed to the article and approved the submitted version.

## Funding

This work was supported by the National Natural Science Foundation of P.R. China (Grant No. 82160168), Shuangqian Project of Scientific and Technological Innovation of High-end Talents-Natural Science, Jiangxi Province (Grant No. jxsq2020101063), and Natural Science Foundation of Jiangxi Province (Grant No. 20212BAB216075).

## Conflict of Interest

The authors declare that the research was conducted in the absence of any commercial or financial relationships that could be construed as a potential conflict of interest.

## Publisher's Note

All claims expressed in this article are solely those of the authors and do not necessarily represent those of their affiliated organizations, or those of the publisher, the editors and the reviewers. Any product that may be evaluated in this article, or claim that may be made by its manufacturer, is not guaranteed or endorsed by the publisher.

## Abbreviations

TG: triglyceride
TC: total cholesterol
HDLC: high-density lipoprotein cholesterol
LDL-C: low-density lipoprotein cholesterol
MCP-1: monocyte chemoattractant protein-1
GSH: glutathione
SOD: superoxide dismutase
CAT: catalase
T-AOC: total antioxidant capacity
GSH-Px: glutathione peroxidase
MDA: malonaldehyde
WAT: white adipose tissue
FFA: free fatty acid
ALT: alanine transaminase
AST: aspartate aminotransferase
IL-1b: interleukin-1b
GTT: glucose tolerance test
ITT: insulin tolerance test
CRP: C-reactive protein
TBARS: thiobarbituric acid reactive substance
eWAT: epididymal white adipose tissue
HOMA-IR: homeostasis model assessment of insulin resistance
NEFA: non-esterified fatty acid
CPT-1: carnitine palmitoyl transferase 1
PDK-4: pyruvate dehydrogenase kinase-4
ABCA1: ATP-binding cassette transporter A1
ROS: reactive oxygen species
IL-8: interleukin-8
FAS: fatty acid synthase
C/EBPα: CCAAT-enhancer binding protein alpha
Ap2: adaptor protein 2.
